# Exploring Neutrophil Extracellular Traps in Canine Soft Tissue Sarcomas: A Preliminary Study With Translational Insights

**DOI:** 10.1002/vms3.71123

**Published:** 2026-07-31

**Authors:** Burak Yildirim, Volkan Ipek

**Affiliations:** ^1^ Health Sciences Institute Burdur Mehmet Akif Ersoy University Burdur Türkiye; ^2^ Faculty of Veterinary Medicine Department of Pathology Burdur Mehmet Akif Ersoy University Burdur Türkiye

**Keywords:** dog, dual immunohistochemistry, extracellular trap, neutrophil, soft tissue sarcoma

## Abstract

**Background:**

Neutrophil extracellular traps (NETs) have gained attention in oncology due to their role in inflammation‐driven tumour progression. While their involvement has been documented in various human malignancies, including select sarcoma subtypes, data on soft tissue sarcomas (STS) remain sparse, particularly in veterinary species.

**Objectives:**

This study aimed to investigate the presence and potential significance of NET formation in spontaneously occurring canine STS, with an additional focus on possible translational relevance to human oncology.

**Methods:**

This study examined 30 canine soft tissue tumours, including fibroma, leiomyoma, fibromyxosarcoma, fibrosarcoma, hemangiosarcoma, liposarcoma, undifferentiated pleomorphic sarcoma, myxosarcoma, perivascular wall tumours, peripheral nerve sheath tumours and undifferentiated sarcoma. NET formations (including both NET‐primed neutrophils and NETs) were detected through a dual‐staining technique using citrullinated histone‐3 (citH3) and myeloperoxidase antibodies on the same tissue sections, employing a fading and restaining method. NET formation was quantified based on citH3‐positive areas and analysed against tumour grade and other histopathological markers.

**Results:**

Pearson correlation analysis found no significant association between NET scores and tumour grade, mitotic activity, necrosis or differentiation. However, NET formation was significantly elevated in malignant sarcomas compared to benign tumours. Within sarcomas, grade III tumours exhibited slightly higher NET formation, though the difference was not statistically significant relative to grades I and II.

**Conclusions:**

These findings parallel emerging evidence in human STS, where NETs have been implicated in tumour progression, though their precise role remains underexplored. By utilizing spontaneous canine sarcomas as a translational model, this study contributes to a deeper understanding of NET‐driven tumour biology and may help potential diagnostic or therapeutic strategies.

## Graphical abstract

Neutrophil Extracellular Traps (NETs) in Canine Soft Tissue Sarcomas

This study investigated the presence of neutrophil extracellular traps (NETs) in canine soft tissue sarcomas using dual immunohistochemistry for citrullinated histone‐3 and myeloperoxidase. Results demonstrated that NET levels were significantly higher in malignant soft tissue sarcomas compared to benign tumours. However, there was no significant correlation between NET scores and tumour grade, necrosis, differentiation or mitosis.

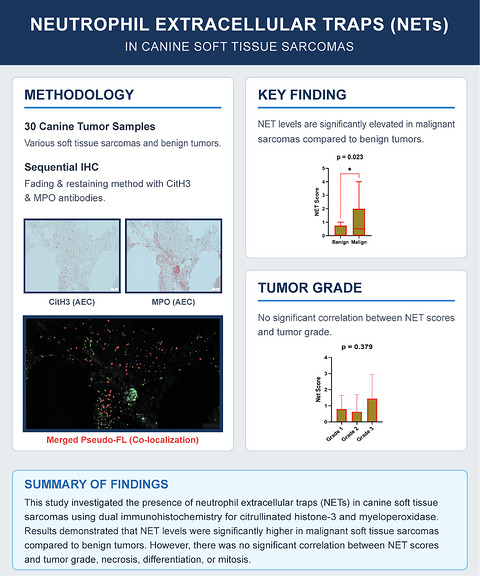

## 1 Introduction

Soft tissue sarcomas are mesenchymal neoplasms originating from soft connective tissues. They can occur in any anatomical region of the body, most commonly seen in areas involving the skin and subcutaneous tissues. STS constitute 8%–15% of the total skin and subcutaneous tumours in dogs and are particularly common among middle‐aged to older, medium to large breed dogs (Vail and Withrow 2001).

These tumours vary in their histological appearance and are named according to the connective tissue (mesenchymal tissue) from which they originate (Ehrhart 2005). Tumours grouped under the STS category include types such as fibrosarcoma, hemangiopericytomas, liposarcoma, rhabdomyosarcoma, leiomyosarcoma, undifferentiated pleomorphic sarcoma (previously termed malignant fibrous histiocytoma), malignant nerve sheath tumours (neurofibrosarcoma, schwannoma), myxosarcoma, myxofibrosarcoma, mesenchymal tumour and spindle cell tumour. Generally, these tumours are low to moderately metastatic and locally invasive (Vail and Withrow 2001; Thrall and Gillette 1995). Other connective tissue‐origin tumours (osteosarcoma, chondrosarcoma, hemangiosarcoma, lymphangiosarcoma and synovial cell sarcoma) are not usually considered STS due to their typically higher metastatic rates (Vail and Withrow 2001; Thrall and Gillette 1995).

Tumour‐associated neutrophils (TANs) constitute a significant portion of immune cells within the tumour microenvironment. High intratumoral neutrophil density is associated with metastasis in lymph node regions. It has been shown that the tumour and the tumour microenvironment control neutrophil recruitment. TANs have been found to regulate tumour progression and control tumour growth (Chen et al. 2021; Masucci et al. 2019). Neutrophil extracellular traps (NETs) consist of condensed chromatin filaments covered with histones and antimicrobial proteins (Ronchetti et al. 2021; Yipp and Kubes 2013). Besides their antimicrobial roles, NETs form a physical barrier for both pathogens and immune cells (Kaltenmeier et al. 2021; Manda‐Handzlik et al. 2020). The NET formation process is classically termed NETosis. NETosis is defined as a regulated type of cell death distinct from apoptosis and necrosis (Manda‐Handzlik et al. 2020). Jung and colleagues (Jung et al. 2019) demonstrated that pancreatic cancer cells could trigger NET formation, which was responsible for increased cell migration, invasion and angiogenesis. In addition, researchers showed that patients with pancreatobiliary tumours had elevated levels of circulating NETs; this was consistent with markers associated with hypercoagulability compared to healthy controls and was associated with disease stage in another study (Miller‐Ocuin et al. 2019). The relationship between circulating NETs and cancer clinical stages has also been reinforced in breast cancer patients (Rivera‐Franco et al. 2020). Overall, these findings indicate that NETs play a dual role in cancer by promoting both the metastatic process and vascular events (Scandolara and Panis 2020).

NETs have emerged as key players in the tumour microenvironment of various human cancers, influencing immune modulation, metastatic spread and tumour‐associated thrombosis (Berger‐Achituv et al. 2013; Schedel et al. 2020; Muqaku et al. 2020; Yao et al. 2023; Yang et al. 2023). However, research on NETs in human STS remains limited. Notably, spontaneously occurring canine STS exhibit striking biological and histopathological similarities to their human counterparts, making them a valuable translational model for cancer research. In this study, we examine the NET formations in canine soft tissue tumours and explore their relationship with tumour grade and other pathological markers. Through this approach, we aim to deepen our understanding of NET‐driven mechanisms in sarcoma, offering potential insights into similar processes in human STS.

## 2 Methods

In the study, 30 blocks of canine soft tissue tumours were used from the department archive (Table 1). This study used archival tissue blocks. According to national regulations (Article 8 of the *Regulation on the Working Procedures and Principles of Animal Experiments Ethics Committees*, Official Gazette Date: 15 February, 2014; no: 28914), formal ethics committee approval is waived for retrospective research strictly involving archived pathology specimens without live animal experimentation. This exemption was officially confirmed by the institutional authority (reference no: [blinded for peer review]). Due to the retrospective use of anonymized archival tissues, the requirement for informed consent from animal owners was waived.

**TABLE 1:** Information on the cases used in the study.

No.		Species	Breed	Gender	Age	Localization	Diagnosis
1.	Dog		Mixed	M	15	—	Fibroma
2.	Dog		Mixed	F	14	Mammary region	Fibroma
3.	Dog		Kopay	M	6	Hip	Fibroma
4.	Dog		Kopay	M	6	Hip	Fibroma
5.	Dog		Russian Toy Terrier	M	10	Gum	Fibroma
6.	Dog		—	—	—	—	Fibroma
7.	Dog		Mixed	F	11	Vagina	Leiomyoma
8.	Dog		Terrier	F	16	Vagina	Leiomyoma
9.	Dog		Mixed	—	1.5	—	Fibromyxosarcoma
10.	Dog		Golden Retriever	M	10	—	Fibromyxosarcoma
11.	Dog		Golden Retriever	M	5.5	Neck	Fibrosarcoma
12.	Dog		Rottweiler	M	4	Right forelimb	Fibrosarcoma
13.	Dog		German Shepherd	M	6	Gum	Fibrosarcoma
14.	Dog		Setter	M	3	Left forelimb	Fibrosarcoma
15.	Dog		Terrier	F	12	Intrathoracic	Fibrosarcoma
16.	Dog		Mixed	F	6	Larynx	Hemangiosarcoma
17.	Dog		Mixed	F	7	Abdominal region	Liposarcoma
18.	Dog		Husky	F	7	Tail base	Liposarcoma
19.	Dog		Rottweiler	M	7	Leg and back	UPS
20.	Dog		Golden Retriever	F	9	Left scapular region	Myxosarcoma
21.	Dog		Mixed	F	17	Axilla	Myxosarcoma
22.	Dog		Mixed	—	—	Perianal region	PWT
23.	Dog		Husky	M	5	Interdigital	PWT
24.	Dog		German Shepherd	M	13	Behind scapula	PWT
25.	Dog		Terrier	M	12	Perianal region	PNST
26.	Dog		Husky	F	15	Tarsal region	PNST
27.	Dog		Mixed	—	—	Mouth edge	PNST
28.	Dog		Mixed	M	6	Lower eyelid	Undifferentiated Sarcoma
29.	Dog		Mixed	M	13	Spleen	Undifferentiated Sarcoma
30.	Dog		Mixed	F	12	Lateral femur	Undifferentiated Sarcoma

Abbreviations: PNST, peripheral nerve sheath tumour; PWT, perivascular wall tumour; UPS, undifferentiated pleomorphic sarcoma.

Samples were placed on normal and lysine‐coated slides. The selection criteria required sufficient tissue for immunohistochemical analysis and a confirmed histopathological diagnosis. Cases with inadequate tissue preservation, insufficient material or ambiguous diagnoses were excluded from the study. The histological classifications of the tumours were performed (Gross 2005; Hendrick 1999; Avallone et al. 2007). According to this, the tumours were classified as fibrous tissue tumours, adipose tissue tumours, muscle tissue tumours, vascular tumours, peripheral nerve sheath tumours and vascular wall tumours. In addition, the tumours were classified as benign and malignant. For the histological grading of malignant tumours, tumour differentiation, mitotic score and tumour necrosis scores were evaluated to determine the final tumour grade.

To detect NET formations in tumour samples, staining was performed with citH3 (citrullinated histone‐3) and myeloperoxidase (MPO) antibodies. A specific method was followed to observe both antibodies in the same tumour sections, which was modified from previous studies (Ezaki 2000; Glass et al. 2009). Initially, sections taken from paraffin blocks for IHC staining were placed on lysine‐coated slides and incubated in a citrate buffer (pH: 6) at 120°C for 15 min in an autoclave. Using a ready immunohistochemistry kit (ImmPRESS Excel Amplified Polymer Staining Kit, Anti‐Rabbit IgG, Peroxidase, MP‐7601, Vector Lab), the citH3 antibody (anti‐histone H3, STJ11100254, St. John's Laboratory, at a 1:100 ratio) was applied overnight. Sections stained with 3‐amino‐9‐ethylcarbazole (AEC, Thermo Scientific, TA‐125‐SA) chromogen were fixed with water‐based adhesive, and photographs were taken under the microscope from designated coordinates at 20× magnification. After photographing, sections were immersed in distilled water to separate the slides and passed through a series of 70%, 95% and 70% ethanol and distilled water to bleach the AEC. Then, to remove the previously bound antibodies and re‐expose the antigens, tissues were boiled again in citrate buffer (pH: 6) in a microwave oven for 2 × 5 min. For the second stage blocking, tissues were incubated with 2.5% normal horse serum (Vectorlab) and, after removing the excess, the MPO antibody (anti‐MPO, STJA0005000, St. John's Laboratory, at a 1:500 ratio) was applied overnight. Sections were re‐stained with AEC, fixed with water‐based adhesive, and photographed again according to the previously taken coordinates. After separating the slides and bleaching the AEC, sections were passed only through distilled water and alcohol series. After nuclear staining with Mayer's haematoxylin (Mayer hemalum, 109249, Merck) for 12 s, preparations were fixed with entellan and photographed again from the same coordinates. Canine suppurative dermatitis tissue, known for its high neutrophil activity and NET formation, was used as a positive control for citH3 and MPO. Negative controls were prepared by replacing the primary antibodies with antibody diluent and isotype‐matched antibodies: mouse IgG2a Kappa for MPO and rabbit IgG for citH3. This approach is supported by previous studies, which used mouse IgG2a and rabbit IgG isotype control antibodies in canine tumours to assess non‐specific staining (Muscatello et al. 2024; Knight et al. 2022). In addition, the specificity of the citH3 antibody (rabbit polyclonal) in canine tissue has been verified by Cherrington and colleagues (Cherrington et al. 2010) using western blot analysis, where a distinct band at approximately 15 kDa, consistent with the expected size of human citH3, was observed. By contrast, verification of MPO by western blot analysis has not been reported in canine tissue; however, an 88.81% amino acid sequence homology between canine and human MPO has been documented in UniProt (Bateman 2019).

### 2.1 Semi‐Quantitative Evaluation

For differentiation, tumours resembling normal adult mesenchymal tissue were scored as 1, tumours with poor differentiation but histologically classifiable were scored as 2 and undifferentiated sarcomas that could not be classified were scored as 3. For mitotic score, 0–9 mitoses were scored as 1, 10–19 mitoses were scored as 2, and >19 mitoses were scored as 3 in 10 high‐power (400×) fields. Finally, for tumour necrosis score, no necrosis was scored as 0, necrosis < 50% was scored as 1, and necrosis > 50% was scored as 2. The final tumour grade was determined by summing the scores of these three parameters. Accordingly, a total score of 3 or less was classified as grade I, 4–5 as grade II and 6 or higher as grade III (Dennis et al. 2011; Trojani et al. 1984).

NET formations in tumour tissues were counted in three separate 20× magnification fields. They were assessed by analysing citH3 and MPO‐positive areas, as well as cells with polymorphonuclear nuclei exhibiting citH3 positivity. To prevent misidentification, eosinophils were confirmed to be absent in haematoxylin‐eosin‐stained sections. In our evaluation, structures exhibiting CitH3 and MPO co‐localization were observed both as intact cells representing NET‐primed neutrophils (early‐stage NETosis) and as scattered free, extracellular, web‐like formations (as shown in Figure 6). The scoring system evaluated both states concurrently to encompass the full NETosis continuum and NETogenic activity within the tumour microenvironment. The density of NET‐associated structures in these areas was scored from 0 to 4 (none, mild, moderate, prominent), similar to our previous study (Karaman and Ipek 2025). To minimize observer bias, the semi‐quantitative evaluation of NET formation was performed independently by the authors of the manuscript, who were blinded to the histological grades. In cases of interobserver discrepancy, a consensus was reached through simultaneous multi‐head microscope review.

### 2.2 Pseudo‐Fluorescent Image Creation

To demonstrate the co‐localization of citH3 and MPO positivity in the same area, images taken from the same coordinates were overlaid in Photoshop (Photoshop CS4 Version 11.0) using the ‘duplicate layer’ option on a haematoxylin‐eosin background. Layers were then slightly adjusted under the ‘difference’ mode to ensure cells matched perfectly. For citH3 and MPO stained sections, the ‘blending mode’ section was set to ‘difference’ mode to colour different stains in red and green. Layers were then merged, and the ‘colour replace’ option was used to darken the background. Finally, the contrast was increased to enhance the background blackness and create pseudo‐fluorescent images.

### 2.3 Statistical Analysis

To determine whether the collected data were normally distributed, the Ryan–Joiner normality test was used. To compare NET scores between benign and malignant tumours, a paired *t*‐test was used. For comparing NET scores by tumour grades, the one‐way ANOVA post hoc Tukey test was applied. In addition, Pearson correlation analysis was performed to examine the relationship between neutrophil and NET scores with tumour characteristics such as mitosis, differentiation and necrosis, as well as with tumour grade. In this analysis, the ‘*r*’ value was considered as insignificant between 0 and 0.3, low between 0.3 and 0.5, moderate between 0.5 and 0.7, high between 0.7 and 0.9, and very high between 0.9 and 1 (Hinkle et al. 2003; Mukaka 2012).

## 3 Results

### 3.1 Histopathological Findings

The diagnoses of the cases used in the study were determined as fibroma (*n* = 6), leiomyoma (*n* = 2), fibromyxosarcoma (*n* = 2), fibrosarcoma (*n* = 5), hemangiosarcoma (*n* = 1), liposarcoma (*n* = 2), undifferentiated pleomorphic sarcoma (*n* = 1), myxosarcoma (*n* = 2), perivascular wall tumour (PWT) (*n* = 3), peripheral nerve sheath tumour (PNST) (*n* = 3) and undifferentiated sarcoma (*n* = 3).

In the histopathological analysis of various tumour samples, distinct features were observed in both benign and malignant cases. Among the benign tumours, fibroma samples exhibited increased fibroblasts with uniform spindle shapes and nuclei, displaying benign characteristics. Similarly, leiomyoma samples showed increased myocytes with prominent pink staining, consistent with benign features. In the malignant tumour samples, fibromyxosarcoma cases demonstrated proliferations of spindle‐shaped cells with malignancy extending in various directions, along with myxoid areas and solid fibrosarcoma regions, with tumour grades ranging from 2 to 3. Fibrosarcoma cases displayed marked differences in cell and nuclear sizes, multinucleated structures and frequent mitotic figures within spindle‐shaped neoplastic proliferations, with tumour grades ranging from 1 to 3.

In hemangiosarcoma, solid areas of spindle‐shaped neoplastic cells with prominent eosinophilic cytoplasm and moderate anisokaryosis were observed, often forming lumens containing a small number of erythrocytes, with a tumour grade of 2. Liposarcoma cases exhibited neoplastic proliferations with numerous cytoplasmic vacuoles and malignancy signs, both determined as grade 2. The undifferentiated pleomorphic sarcoma sample revealed proliferations of spindle‐shaped mesenchymal cells with marked anaplasia, including numerous multinucleated giant cells, graded as 3. Myxosarcoma cases showed spindle‐shaped proliferations with extensive mucinous material and significant nuclear size differences, graded as 2 and 3.

**FIGURE 1:**
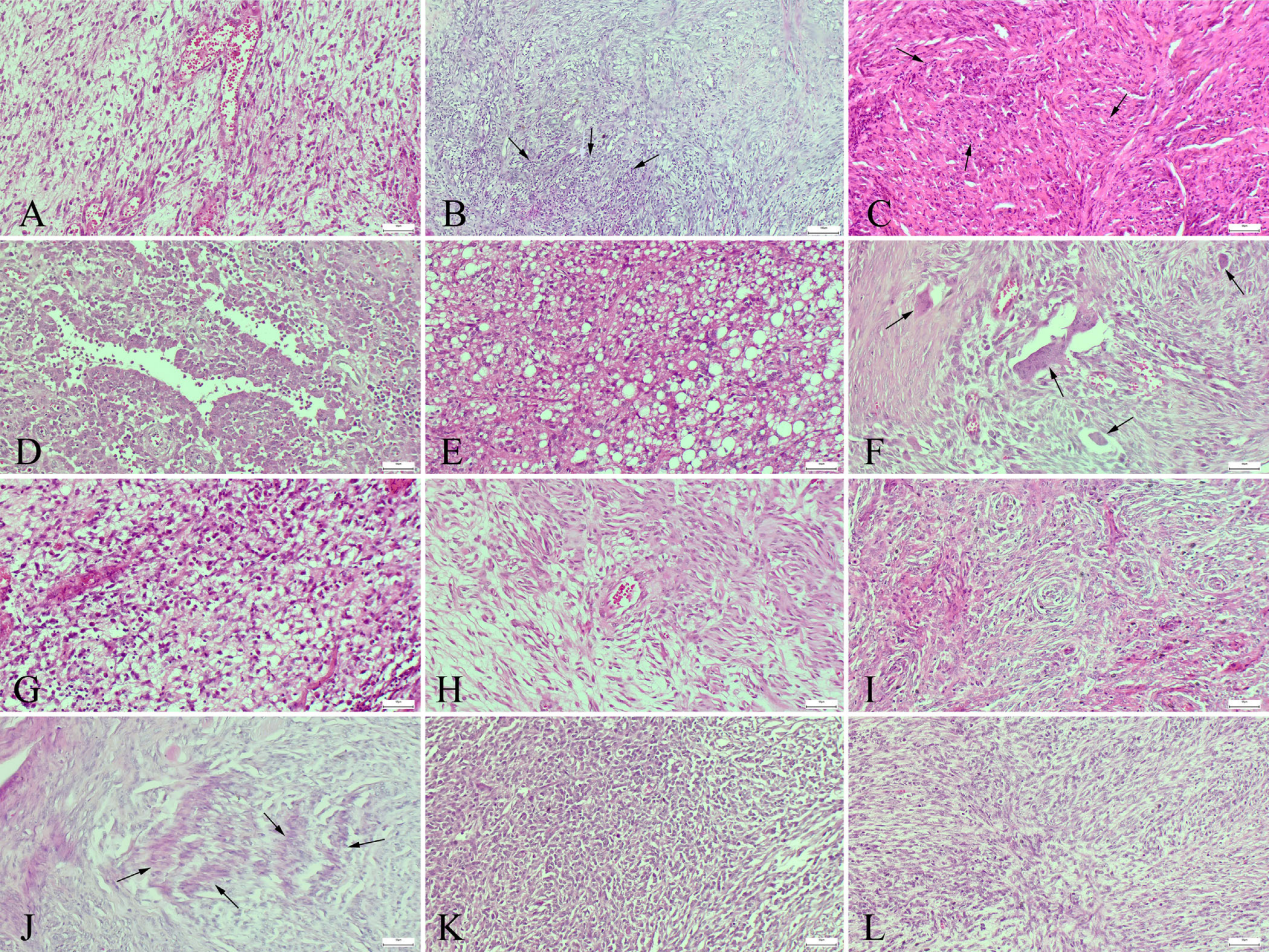
Combined neoplastic features in various tumour cases. H&E. Bar: 50 µm (unless otherwise stated). This figure illustrates a range of neoplastic characteristics observed in different tumour types. (A) shows neoplastic proliferations with myxomatous formations and signs of malignancy in a fibromyxosarcoma case (Case 10). (B) highlights a fibromyxosarcoma area with marked atypia and intense neutrophil infiltrations (arrows), observed at a magnification with a bar of 100 µm (Case 9). (C) depicts a neoplastic area with prominent eosinophilic cytoplasm and malignancy, with scattered neutrophils in the tumour tissue (arrows) in a fibrosarcoma case (Case 12). (D) presents endothelial proliferations with a tendency to form vascular lumens and prominent eosinophilic cytoplasm in a hemangiosarcoma case (Case 16). (E) shows a neoplastic area with numerous intracytoplasmic vacuoles and prominent malignant nuclear features (case 18). (F) illustrates the proliferation of connective tissue with numerous multinucleated giant cells (arrows) in a undifferentiated pleomorphic sarcoma case (Case 19). (G) demonstrates a highly cellular tumour area with malignancy features in a myxosarcoma case (Case 21). (H) reveals a neoplastic area with a prominent myxoid appearance (star) and proliferation around vessels in a PWT case (Case 24). (I) features a neoplastic area with prominent malignancy features and spiral structures around vessels in a PWT case (Case 22). (J) depicts a neoplastic appearance with an Antoni A type pattern (arrows) in a PSKT case (Case 27). (K) highlights mesenchymal proliferations with poor differentiation and prominent malignancy features in an undifferentiated sarcoma case (Case 28), and (L) shows a tumour area with prominent malignancy features and an irregular anaplastic appearance in another undifferentiated sarcoma case (Case 29).

In PWT cases, neoplastic cells were observed proliferating spirally around central vessels, with one case exhibiting a prominent myxoid appearance graded as 3, another with mild malignancy signs graded as 1, and a highly cellular case with severe malignancy graded as 3. Malignant PNST cases displayed prominent spiral structures without central vessels and interwoven bundles with Antoni A and B type patterns, graded as 1 and 2. Finally, undifferentiated sarcoma cases showed poorly differentiated, spindle‐shaped, pleomorphic proliferations with varying nuclear sizes and numerous mitotic figures. Representative images of all cases are presented in Figure 1.

### 3.2 Immunohistochemical Findings

In the examinations for NET formation, only one of the fibroma cases had a score of 1, while the other five cases were determined to be 0. In the leiomyoma cases, one had a score of 0, and the other was 1. Among the fibromyxosarcoma cases, one had a score of 1, and the other had a score of 4. For the fibrosarcoma cases, two had a score of 0, one had a score of 1 and two had a score of 2. The hemangiosarcoma case had a score of 3. The liposarcoma cases had a score of 0, and the undifferentiated pleomorphic sarcoma case had a score of 2. One of the myxosarcoma cases had a score of 0, and the other was 2. In the PWT cases, two had a score of 0, and one had a score of 3. Among the PSKT cases, one had a score of 0, and the others had a score of 1. All undifferentiated sarcoma cases had a score of 0. Information on NET scores and histological grading scores of the tumours is presented in Table 2.

**TABLE 2:** Diagnosis and scores of cases.

No.	Diagnosis	NETs	Mitosis	Differentiation	Necrosis	Grade
1.	Fibroma	0	—	—	—	—
2.	Fibroma	0	—	—	—	—
3.	Fibroma	0	—	—	—	—
4.	Fibroma	1	—	—	—	—
5.	Fibroma	0	—	—	—	—
6.	Fibroma	0	—	—	—	—
7.	Leiomyoma	0	—	—	—	—
8.	Leiomyoma	1	—	—	—	—
9.	Fibromyxosarcoma	1	1	2	1	2
10.	Fibromyxosarcoma	4	2	3	1	3
11.	Fibrosarcoma	0	1	2	1	2
12.	Fibrosarcoma	2	2	1	0	1
13.	Fibrosarcoma	1	1	1	1	1
14.	Fibrosarcoma	0	1	2	1	2
15.	Fibrosarcoma	2	3	2	1	3
16.	Hemangiosarcoma	3	1	3	0	2
17.	Liposarcoma	0	1	2	1	2
18.	Liposarcoma	0	1	2	1	2
19.	UPS	2	3	3	2	3
20.	Myxosarcoma	0	3	1	1	2
21.	Myxosarcoma	2	2	2	2	3
22.	PWT	0	3	2	1	3
23.	PWT	0	1	2	0	1
24.	PWT	3	1	3	2	3
25.	PNST	0	1	2	0	1
26.	PNST	1	1	2	1	2
27.	PNST	1	1	2	0	1
28.	Undifferentiated sarcoma	0	3	3	0	3
29.	Undifferentiated sarcoma	0	2	3	1	3
30.	Undifferentiated sarcoma	0	2	3	1	3

*Note*: **—** indicates parameters not evaluated for benign tumours.

Abbreviations: PNST, peripheral nerve sheath tumour; PWT, perivascular wall tumour; UPS, undifferentiated pleomorphic sarcoma.

**FIGURE 2:**
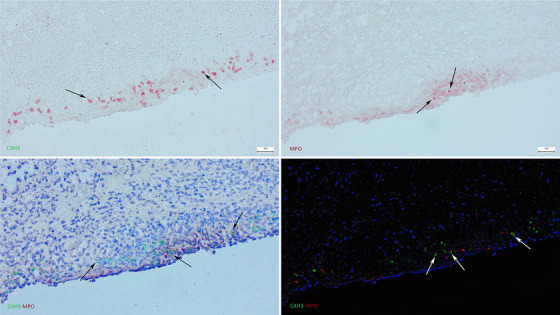
Overlay of CitH3 and MPO positivity (AEC chromogen) on a haematoxylin‐stained background in a fibromyxosarcoma (Case 9), revealing co‐localized signals indicative of NET formation (arrows). The pseudo‐immunofluorescent appearance highlights areas of intense staining. Scale bar: 20 µm.

**FIGURE 3:**
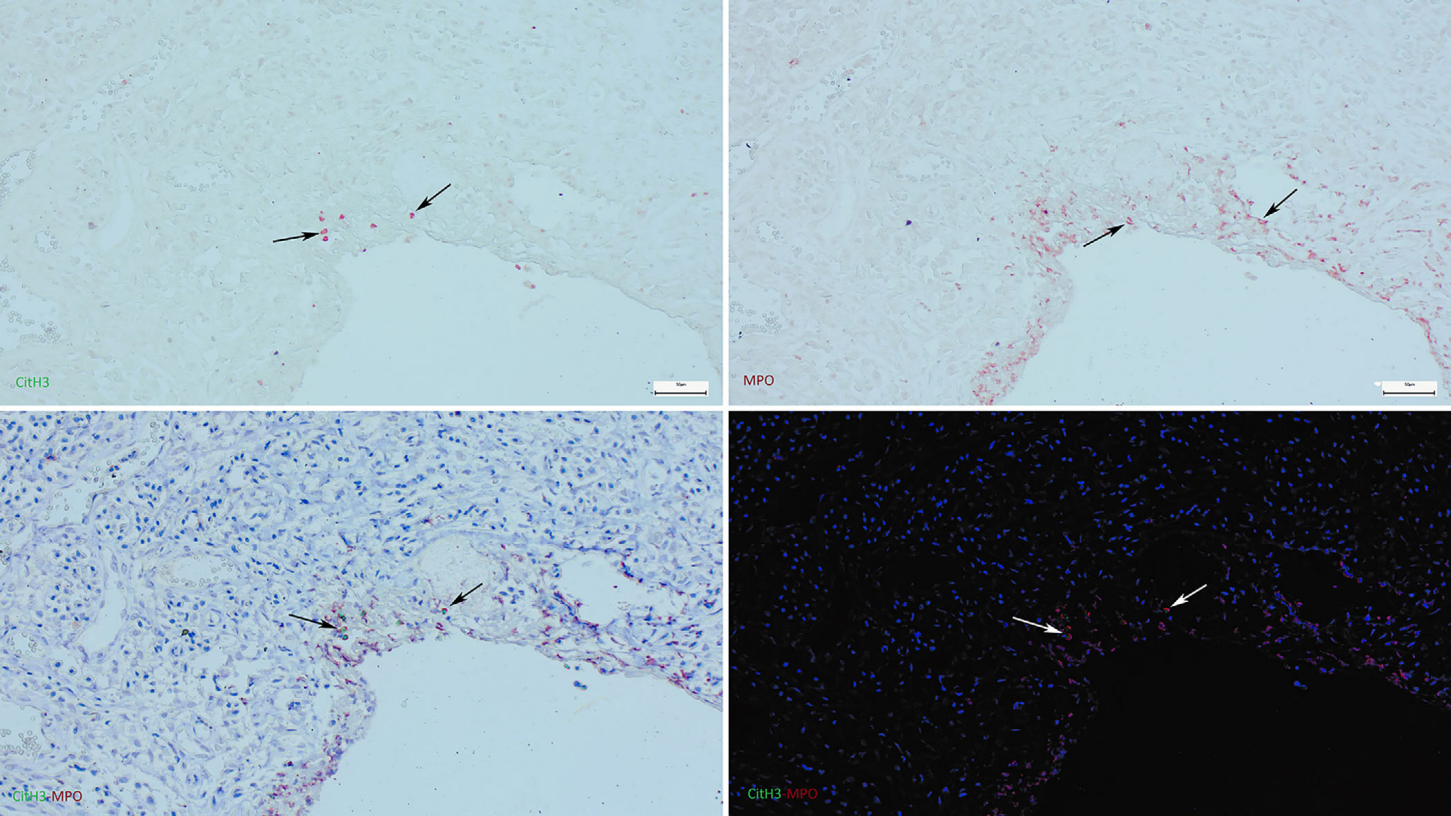
Merged visualization of CitH3 and MPO expression in a hemangiosarcoma (Case 16), demonstrating prominent co‐positivity in tumour‐associated neutrophils (arrows). The pseudo‐immunofluorescent effect enhances contrast for better localization. Scale bar: 50 µm.

**FIGURE 4:**
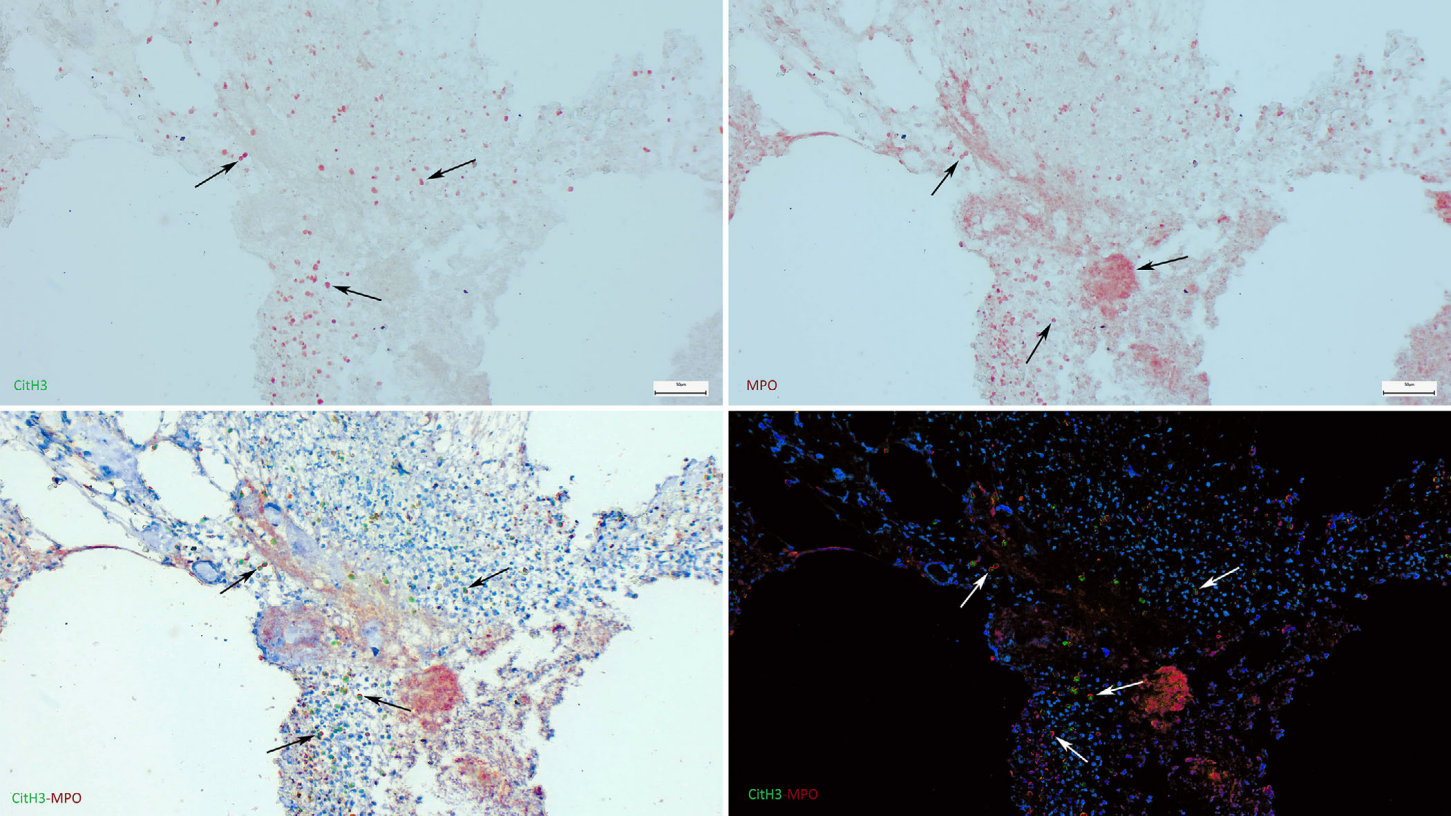
Superimposed CitH3 and MPO staining in an undifferentiated pleomorphic sarcoma (Case 19), showing widespread neutrophil involvement and NET structures (arrows). The pseudo‐immunofluorescent technique enhances marker distribution. Scale bar: 50 µm.

**FIGURE 5:**
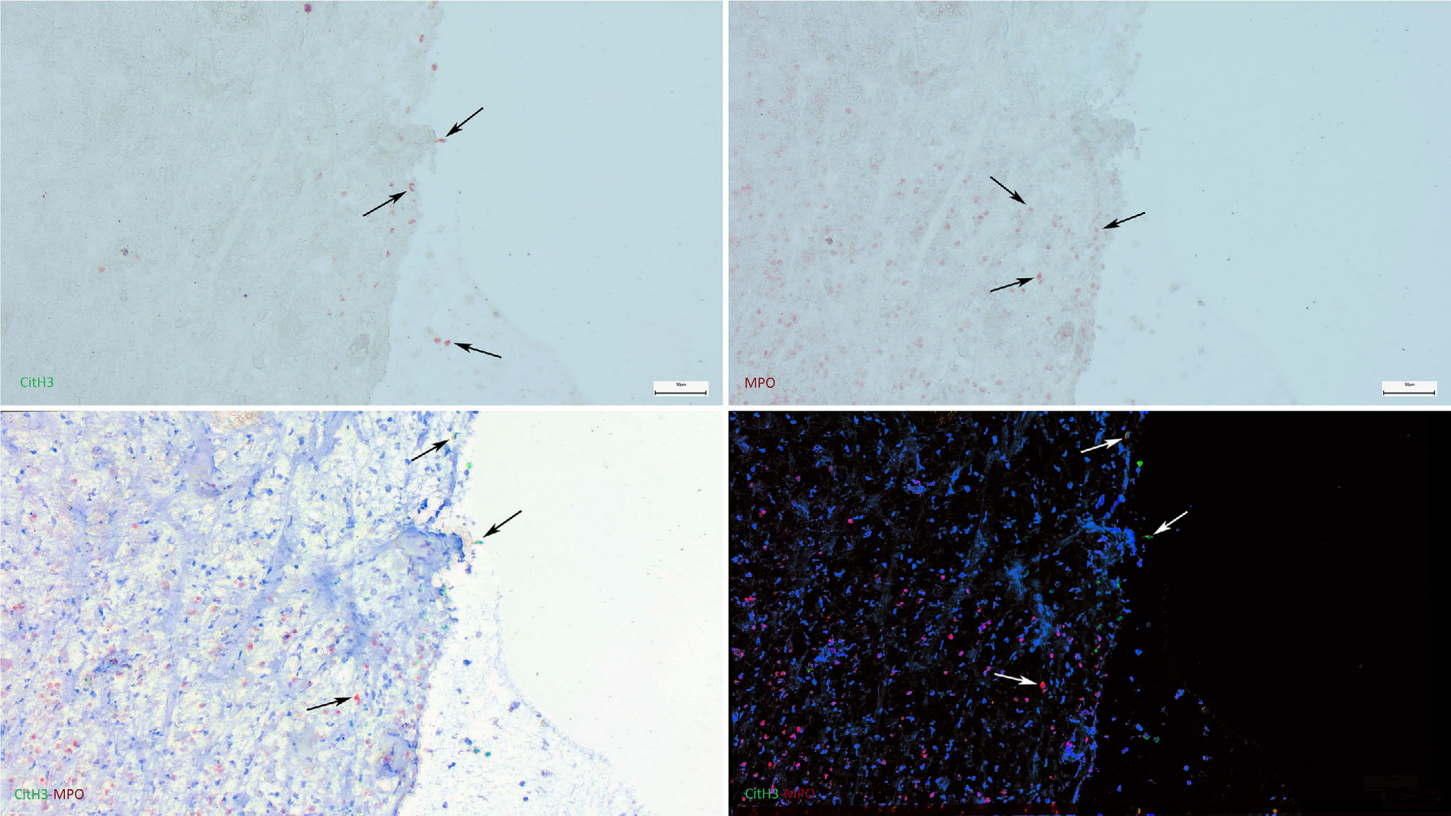
Composite image of CitH3 and MPO staining in a myxosarcoma (Case 21), illustrating distinct regions of neutrophil activation and extracellular trap formation (arrows). The pseudo‐immunofluorescent representation aids visualization. Scale bar: 50 µm.

**FIGURE 6:**
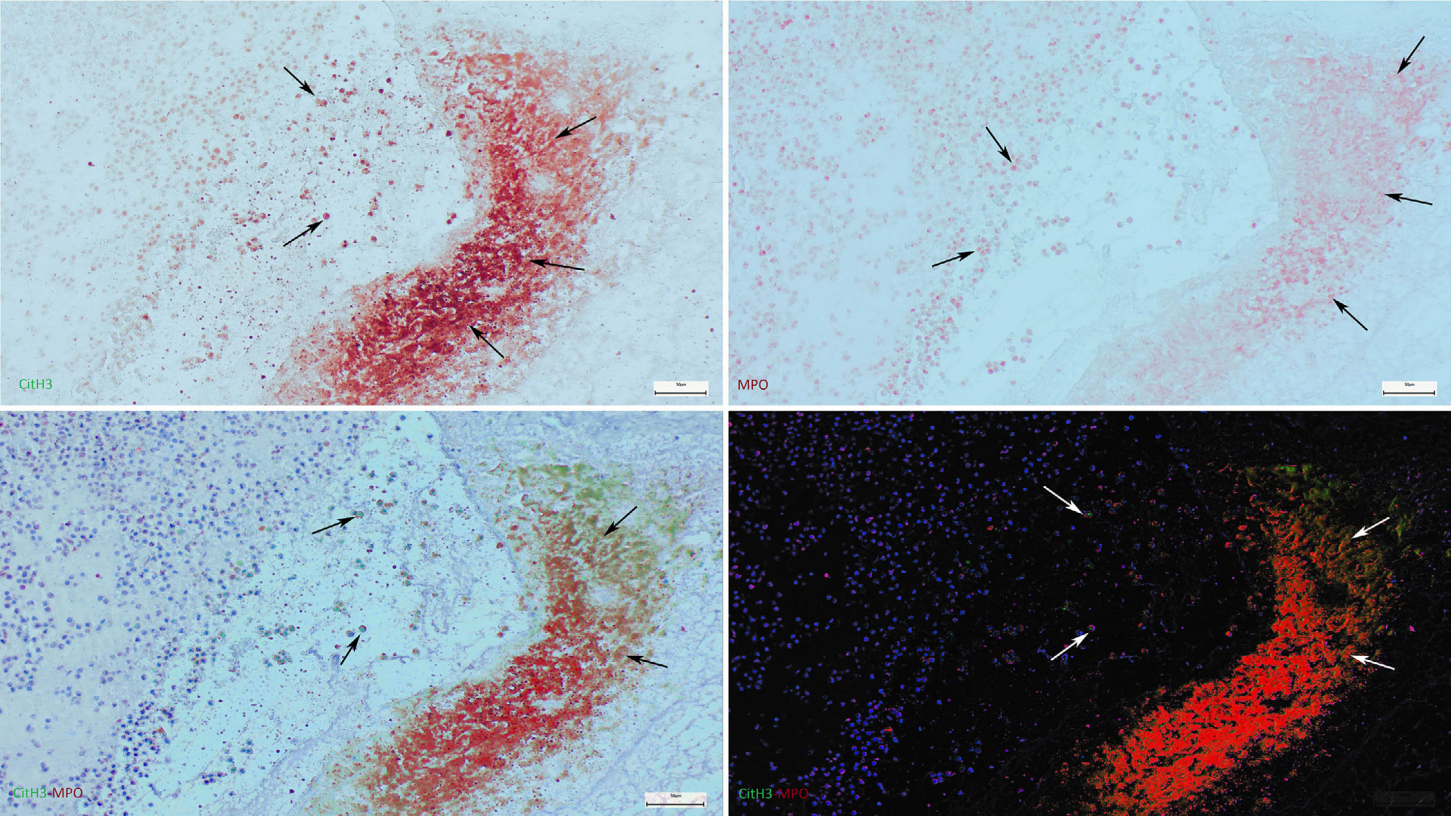
Visualization of CitH3 and MPO expression in a PWT (Case 24), depicting co‐localization patterns associated with NET presence (arrows). The pseudo‐immunofluorescent method emphasizes structural details. Scale bar: 50 µm.

**FIGURE 7:**
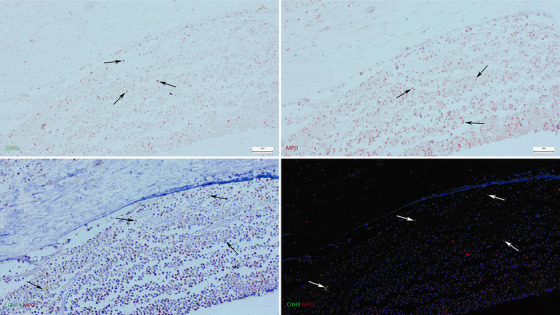
Reconstructed overlay of CitH3 and MPO staining in another section of the same PWT (Case 24), highlighting regions of NET activity with distinct co‐localization (arrows). The pseudo‐immunofluorescent processing enhances clarity. Scale bar: 50 µm.

Images from the immunohistochemical staining and the resulting photos after merging and colouring are shown in Figures 2, 3, 4, 5, 6, 7.

### 3.3 Statistical Analysis Results

In the paired *t*‐test conducted to compare NET formation in benign and malignant tumours, it was found that NET scores were significantly higher in malignant tumours compared to benign ones (*p* = 0.023) (Table 3).

**TABLE 3:** Comparison of NET scores in benign and malignant tumours.

Tumour	NET
Benign	0.0 (0.0–0.75)
Malignant	0.5 (0.0–2.0)

*Note*: Values are presented as median (IQR). Malignant tumours show significantly higher NET scores.

Cases were grouped according to tumour grade, and the data in the groups were found to be normally distributed. Then, statistical comparisons were made between the groups using the one‐way ANOVA test with Tukey analysis, and it was found that although there was a numerical increase in NET scores in grade III tumours compared to grade I and II tumours, there was no significant difference (*p* = 0.379). The statistical data are presented in Table 4.

**TABLE 4:** Comparison of necrosis, neutrophil, and NET scores by tumour grade.

Tumour grade	NET
Grade I	0.800 ± 0.374
Grade II	0.625 ± 0.375
Grade III	1.444 ± 0.503

*Note*: Values are presented as mean ± standard deviation. NET scores are higher in grade III tumours.

In the Pearson correlation analysis conducted to examine the relationship between NET score and mitosis, differentiation, necrosis and tumour grade, it was determined that the non‐significant positive correlation between NET score and tumour grade (*r* = 0.277). Similarly, the correlations between NET score and mitosis, differentiation, and necrosis were also found to be non‐significant. The statistical results are presented in Table 5.

**TABLE 5:** Pearson correlation analysis results.

		NET	Mitosis	Differentiation	Grade
Mitosis	*r* value *p* value	0.046 0.839			
Differentiation	*r* value *p* value	0.290 0.190	0.109 0.630		
Necrosis	*r* value *p* value	0.241 0.279	0.182 0.419	0.173 0.441	
Grade	*r* value *p* value	0.243 0.277	0.591 0.004	0.656 0.001	0.613 0.002

*Note: r* value—Pearson correlation coefficient, indicating the strength and direction of the relationship between variables. *p* value—statistical significance; values < 0.05 are considered significant. Non‐significant positive correlation was observed between Grade and NET.

## 4 Discussion

Neutrophils perform the process of NETosis, which involves the release of extracellular web‐like structures called NETs, in addition to phagocytosis and degranulation to exert their antimicrobial activities (Ronchetti et al. 2021; Burgener and Schroder 2020; Papayannopoulos 2018). Besides their antimicrobial roles, NETs also form a physical barrier for both pathogens and immune cells (Kaltenmeier et al. 2021; Manda‐Handzlik et al. 2020). NETs are web‐like structures made of nuclear or mitochondrial DNA fibres containing antimicrobial enzymes and histones released to capture and kill pathogens (Kaltenmeier et al. 2021; Pruchniak and Demkow 2019). NETs consist of condensed chromatin filaments coated with histones and antimicrobial proteins (Ronchetti et al. 2021; Yipp and Kubes 2013). They contain proteins such as neutrophil elastase, MPO, cathepsin G, proteinase 3, lactoferrin, gelatinase, lysozyme C, calprotectin, neutrophil defensin and cathelicidin (Korkmaz et al. 2008; Kwak et al. 2022). Histone citrullination and its role in the NETosis process are significant areas of study in immunology, particularly in connection with various diseases. Histone citrullination involves the modification of histone tails in activated neutrophils, a process crucial for the formation of NETs. NET formation is closely associated with the activation of peptidyl arginine deiminase 4 (PAD4), an enzyme that converts arginine to citrulline and is considered an indicator of NETosis (Neeli et al. 2008; Leshner et al. 2012). MPO also plays a significant role in the NETosis process. MPO is an intracellular factor released by neutrophils and found in DNA‐containing structures (Dyer et al. 2018). In light of this information, CitH3 and MPO, commonly used markers for NETs, were used to demonstrate the NET formation.

In our previous study (Karaman and Ipek 2025), we used the method of staining the same tissue section with CitH3, then bleaching and antigen retrieval, followed by staining with MPO in canine mammary cancer. This method was performed by adding the heat‐induced antigen retrieval process used in other studies to the method described by Ezaki (2000), which involves acidic potassium permanganate application (Glass et al. 2009). Our previous study also confirmed that the combined use of these two methods effectively dissolved antibodies. However, in this study, we removed the acidic potassium permanganate application and used only the heat‐induced antigen retrieval in citrate buffer, which also effectively removed previous antibody bindings.

The bleaching and re‐staining method can lead to tissue peeling, and taking images from the same region is more labour‐intensive compared to dual immunohistochemistry or immunofluorescence methods. However, in this study, it was observed that tissue peeling was minimal, and overall tissue integrity was well preserved up to the final haematoxylin‐stained preparations. It is believed that removing the acidic potassium permanganate application further reduced the peeling. The applicability of this method has been confirmed by using numerous antibodies, with reports of applying up to 12 different stains on the same sections (Tsujikawa et al. 2017). As previously demonstrated, the method's ability to use numerous antibodies makes it a highly useful method for formalin‐fixed paraffin block sections, where fluorescent staining can be problematic.

In our previous study on canine soft tissue tumours, neutrophils were not associated with malignancy but might play a role in the increase of macrophage infiltrations (Savaş and İpek 2021). In another study (Karaman and Ipek 2025) on canine mammary tumours, where we examined the NET formation using the same method, a non‐significant negative correlation (*r* = −0.219) was observed between NET scores and tumour grade. In the current study, the NET formation was significantly higher in STS compared to benign tumours. Furthermore, when considering the histological grading of sarcomas, the presence of NETs was slightly higher in grade III tumours, but this difference was not statistically significant compared to other grades. In addition, NET scores had an insignificant correlation with necrosis, differentiation, mitosis and tumour grade in sarcomas.

Our findings reveal a significant disparity in NET formation between benign and malignant canine soft tissue tumours, suggesting a potential link between NETs and tumour progression. These results align with preliminary human studies—most notably, a small prospective analysis of Ewing's sarcoma—where intratumoral NETs were associated with a higher risk of metastasis and disease recurrence (Berger‐Achituv et al. 2013). While the limited sample size of that study prevents definitive conclusions, it underscores the possibility that NETs play a pivotal role in STS biology. In addition, recent machine learning–driven research on NET‐related lncRNAs in human STS further highlights NETs as potential prognostic markers or therapeutic targets (Liu et al. 2023).

Given the well‐documented similarities between canine and human STS in histopathology, clinical behaviour and immune interactions, our canine‐based findings could serve as a translational foundation for future research into NETs’ role in human sarcomas. Canine models offer key advantages over traditional rodent models, including spontaneous tumour development, an intact immune system, and genetic diversity, all of which more closely replicate the complexity of human tumour biology (Vail and MacEwen 2000; Klosowski et al. 2023). Given the scarcity of data on NETs in human STS, larger‐scale comparative studies in both canine and human cohorts are needed to determine whether targeting NETs or their upstream regulators—such as PAD4 inhibitors—could help mitigate metastatic potential and reduce the risk of early relapse.

This study has certain limitations. First, the relatively small sample size may have influenced the statistical significance of correlation analyses. Future studies with larger cohorts are needed to validate these findings. In addition, as this is a retrospective study using archival samples, clinical follow‐up data were unavailable, preventing an assessment of the relationship between NET abundance and clinical outcomes. Finally, the potential role of circulating NETs as biomarkers remains unexplored. Future research should investigate their presence in plasma and their correlation with tumour progression to determine their diagnostic and prognostic value.

## 5 Conclusion

Our study provides the first evidence of NET formation in canine STS, offering valuable insights that may help bridge existing knowledge gaps in human STS. Given the substantial histopathological and biological similarities between spontaneous canine sarcomas and their human counterparts, these tumours serve as a promising translational model for exploring NET‐driven mechanisms. Future research on NET‐based biomarkers and targeted therapies in canine models could ultimately inform the development of more effective diagnostic and treatment strategies for both veterinary and human STS.

## Author Contributions

**Burak Yildirim**: data curation, formal analysis, investigation, methodology, visualization, and writing – original draft. **Volkan Ipek**: conceptualization, formal analysis, funding acquisition, investigation, methodology, project administration, resources, supervision, validation, writing – original draft, and writing – review and editing.

## Funding

This research was supported by the Scientific Research Projects Coordination Unit of Burdur Mehmet Akif Ersoy University (Project number: 0905‐YL‐23).

## Ethics Statement

This study was conducted strictly using archival tissue blocks from the Department of Pathology. According to Article 8 of the Regulation on the Working Procedures and Principles of Animal Experiments Ethics Committees of the Republic of Turkey (Official Gazette Date: 15 February, 2014; no: 28914), formal ethics committee approval is not required for studies utilizing archived pathology specimens without any experimental procedures performed on live animals. This exemption was officially confirmed by the Burdur Mehmet Akif Ersoy University (Reference no: E‐93773921‐020‐523248; Date: 16 May, 2025). Furthermore, due to the retrospective nature of the study and the use of anonymized archival tissues, the requirement for written informed consent from the animal owners was waived.

## Conflicts of Interest

The authors declare no conflicts of interest.

## Data Availability

The data that support the findings of this study are available from the corresponding author upon reasonable request.
